# Interstitial lung disease in infancy and early childhood: Clinical approach

**DOI:** 10.1002/ppul.27254

**Published:** 2024-09-11

**Authors:** Andrew Bush

**Affiliations:** ^1^ National Heart and Lung Institute, Imperial College, and Imperial Centre for Paediatrics and Child Health London UK; ^2^ Department of Paediatric Respiratory Medicine Royal Brompton Hospital London UK

**Keywords:** alveolar capillary dysplasia, ECMO, lung biopsy, NEHI, pulmonaty interstitial glycogenosis, surfactant protein

Early onset interstitial lung disease in children (chILD) presents in one of two ways. The first is in a term baby with unexpected and rapidly progressive respiratory failure, requiring intubation and often ECMO. The second, later‐presenting is subacute, either with respiratory symptoms (tachypnoea, increased work of breathing, cyanosis) or more nonspecifically, with feeding difficulties and failure to thrive. The diagnostic pathway differs; the acutely deteriorating baby may be too sick to move to the CT scanner and proceeds straight to genetic testing and even lung biopsy to get a diagnosis (below). With a more indolent presentation, CT scan confirms the presence of chILD rather than commoner issues such as aspiration. The CT may be able to predict the lung histology, for example, neuroendocrine cell hyperplasia of infancy (NEHI, below). Further investigations including blood tests for genetic analysis and autoantibodies are next performed, and if no diagnosis can be made, lung biopsy is considered. Bronchoscopy is rarely performed as a single procedure; bronchoalveolar lavage is rarely diagnostic, and the samples obtained at transbronchial biopsy are usually too small for a confident diagnosis, and the risk is not trivial. The aim of investigations is to find a specific cause in one of four categories (Table 1). In early chILD, genetic and unknown causes predominate, reviewed in detail in.^1^

**Table 1:** “Four‐box” approach to the classification of chILD.

Underlying category	Comments	Treatment approach
Genetic	Definite: disease causing mutation	Understand sub‐endotypes
	Probable/possible: VUS, +ve family history	Aim for specific therapies
Environmental	Detailed history vital	Remove stimulus +/steroids
	Includes vaping	
Other known nongenetic	For example, iatrogenic, GMCSF autoantibodies	Remove cause if possible
		Systemic steroids
		May be specific therapies for example, inhaled GMCSF
As yet unknown cause	Fruitful area for research	Only nonspecific treatments possible

Abbreviations: GMCSF, granulocyte‐macrophage colony stimulating factor; VUS, variants of unknown significance.

## 1 EARLY‐ONSET ACUTE CHILDS

The commonest causes are the congenital alveolar dysplasia–alveolar capillary dysplasia spectrum (CAD‐ACD), and surfactant protein (Sp) variants, especially *SpB* and *ABCA3*, but also *SpC*. In particular, ACD with misaligned pulmonary veins (ACDMPV) usually presents with rapid deterioration needing the institution of ECMO soon after birth. The commonest underlying genetic cause is *FOXF1* disease, but this is a complex gene, and many disease‐causing variants are in the promotor region. This may result in the genetic diagnosis being delayed, in which case, an early lung biopsy can establish a histopathological diagnosis and allow withdrawal of care instead of prolonged invasive and distressing treatments. Even on ECMO, a lung biopsy can usually be obtained safely and the diagnostic yield is higher than in older children.^2^ Sp disease is usually more slowly progressive, but also relentless*. ABCA3* with two severe variants presenting in the newborn period leads to death or transplantation within a year. An *SpB* mutation cannot be detected in 30% of babies with absent SpB immunostaining on biopsy; it may be that some of these represent mutations in genes involved in the complex posttranslational processing of SpB. There is now an expanding number of genetic defects of Sp metabolism and catabolism being described. Premature delivery leading to bronchopulmonary dysplasia (BPD) does not exempt the baby from also having a chILD, and referrals of babies with end stage BPD are frequently made to exclude chILD; the CT appearances should be distinctive, although occasionally some of these children are biopsied.

## 2 SUBACUTE EARLY CHILDS

The classical diseases are NEHI and pulmonary interstitial glycogenosis (PIG). CT scanning of NEHI classically reveals ground glass shadowing in the middle lobe and lingula and in the perihilar regions, sparing the periphery. Biopsy is not usually necessary, but if performed, shows increased numbers of distal airway bombesin positive cells. NEHI is a description not a diagnosis and has genetic and likely other underlying causes.^3^ The treatment is supportive, but the child may need supplemental oxygen for several years. PIG is characterised by mesenchymal glycogen positive cells (it is unrelated to glycogen storage diseases, there is no systemic glycogen deposition). It is frequently associated with alveolar hypoplasia and growth disorders. Although some report a response to steroids, that is not my experience. Prognosis is good, but deaths are recorded in preterm babies with PIG. Sp mutations (*SpC*, *ABCA3*, *NKX2.1*) also present sub acutely in this age group, as well as the other disorders of Sp metabolism and catabolism. An increasing number of genetic chILDs are being described, including filamin and integrin mutations, interferonopathies (SAVI) and COPA mutations, all of which may have extrapulmonary manifestations, unlike *SpB*, *SpC* and *ABCA3* disease (alone among Sp mutations, NXK2.1 sometimes manifests with extrapulmonary disease, affecting the brain and thyroid).

## 3 THE CHANGING CHILD DIAGNOSTIC PATHWAYS

Lung biopsy is no longer the end of the diagnostic journey, and increasing numbers of children are being diagnosed on genetic testing. Biopsy is not a gold standard. The same morphological pattern (e.g., pulmonary alveolar proteinosis) may be caused by many different genetic mutations (Sp metabolism and catabolism, MARS, and GATA‐2 e.g.). The same genetic mutation can cause multiple different biopsy abnormalities, and the biopsy findings for a given mutation may change over time if the individual is re‐biopsied. However the biopsy may point the way to further investigations for example, granulomatous‐lymphocytic ILD pointing to immunodeficiency. The biopsy may also suggest the need for specific genetic studies. Unless the child is deteriorating rapidly, genetic and other serological tests would be obtained before proceeding to biopsy. A recent large study reporting the safety of lung biopsy showed that an elective procedure should have a mortality of <1%, nonelective procedures, age <3 months and comorbidity, especially if the technique is not video‐assisted, carry a greater risk.^4^

## 4 THE CHANGING APPROACH TO TREATMENT OF CHILD

For most chILDs, only nonspecific empirical therapies such as prednisolone, azithromycin and hydroxychloroquine are available. However, the point of making the diagnosis of a rare genetic condition is that highly specific and effective treatments are available, for example anti‐IL6 monoclonals such as tocilizumab and sarilumab combined with JAK inhibitors, for example ruxolitinib, baricitinib, tofacitinib, and Upadacitinib.

## 5 CONCLUSIONS

Genetic testing is becoming increasingly important in the diagnosis of chILD; many new entities are being discovered and many more will be discovered in the next few years. Lung biopsy is becoming less important in the diagnostic pathway, but has a definite role, especially in the rapidly deteriorating child in whom no diagnosis has been made. Genetic testing opens up the possibility of personalised medicine for chILD, and this is a growth area, including for the deployment of small molecule therapy similar to those which have transformed the prognosis of cystic fibrosis.^5^

## AUTHOR CONTRIBUTIONS

**Andrew Bush**: Conceptualization; investigation; writing—original draft; writing—review and editing.

## CONFLICT OF INTEREST STATEMENT

The author declares no conflicts of interest.

## Data Availability

The data that support the findings of this study are available from the corresponding author upon reasonable request.
